# Analysis of Contact Position for Subthalamic Nucleus Deep Brain Stimulation-Induced Hyperhidrosis

**DOI:** 10.1155/2019/8180123

**Published:** 2019-03-06

**Authors:** Chunhui Yang, Yiqing Qiu, Xi Wu, Jiali Wang, Yina Wu, Xiaowu Hu

**Affiliations:** Department of Neurosurgery, Second Military Medical University, Changhai Hospital, No. 168 Changhai Road, Yangpu District, Shanghai, China

## Abstract

**Objectives:**

To analyze the hyperhidrosis neural network structure induced by subthalamic nucleus (STN) - deep brain stimulation (DBS).

**Materials and Methods:**

Patients with Parkinson's disease treated with STN-DBS in Changhai Hospital between July 1, 2015, and December 1, 2016, were analyzed retrospectively. Using records of side effects of the intraoperative macrostimulation test, patients with skin sweats were selected as the sweating group. Based on the number of cases in the sweating group, the same number of patients was randomly selected from other STN-DBS patients without sweating to form the control group. The study standardized electrode position with Lead-DBS software to Montreal Neurological Institute (MNI) standard stereotactic space to compare the differences in three-dimensional coordinates of activated contacts between groups.

**Results:**

Of 355 patients, 11 patients had sweats during intraoperative macrostimulation tests. There was no significant difference in the preoperative baseline information and the postoperative UPDRS-III improvement rate (Med-off, IPG-on) between groups. Contacts inducing sweat were more medial (*X*-axis) (11.02 ± 0.69 mm vs 11.98 ± 0.84 mm, *P*=0.00057) and more upward (*Z*-axis) (−7.15 ± 1.06 mm VS −7.98 ± 1.21 mm, *P*=0.032) than those of the control group. The straight-line distance between the center of the sweat contact and the nearest voxel of the red nucleus was closer than that of the control group (2.72 ± 0.65 mm VS 3.76 ± 0.85 mm, *P*=0.00012).

**Conclusions:**

STN-DBS-induced sweat indicated that the contact was at superior medial of STN.

## 1. Introduction

Subthalamic nucleus (STN) and globus pallidus internus (GPi) deep brain stimulation (DBS) has substantial effects on idiopathic Parkinson's disease (PD) [1]. Some authors found that zona incerta (Zi) DBS [2] and prelemniscal radiation (Raprl) DBS [3] are also options for treating PD; posterior subthalamic area- (PSA-) DBS treats essential tremor [4] and medication-refractory tremor of PD [5, 6]. However, STN-DBS [7] or PSA-DBS may lead to sweating for some patients [8, 9].

The position of contacts of PSA-DBS and their effect on sweat was explored [10]. However, there are two problems not resolved by previous studies. (1) Due to the small number of patients in previous studies, patients' imaging data were not uniformized. Therefore, the anatomical positions of active contacts based on AC-PC are diverse. (2) Sweating was observed only during STN-DBS, and active contacts did not reach PSA. This lead to the question of whether there were any other anatomical structures related to sweat.

To address above two questions, the current study standardized enough patient's brain imaging data into the three-dimensional space of Montreal Neurological Institute (MNI) to determine that an active contact anatomical position of STN-DBS-induced sweating.

## 2. Materials and Methods

### 2.1. Data of Patients

The study acquired approval from Shanghai Changhai Hospital Ethics Committee. Patients with PD treated with STN-DBS in Shanghai Changhai Hospital between July 1, 2015, and December 1, 2016, were analyzed retrospectively. Patients met the diagnostic criteria of PD UK Brain Bank [11], and the surgical indications met Expert Consensus of DBS for the Treatment of Parkinson's Disease in China [12]. While data mining all intraoperative electrical stimulation tests record, patients with sweats were selected as the sweating group. A same number of matched patients were selected from other patients as the control group to compare differences in position of activated contacts.

### 2.2. Imaging and Operation

#### 2.2.1. Preoperative Magnetic Resonance and CT

All cases underwent preoperative MR imaging on a 3.0 T scanner (MAGNETOM Skyra, Siemens, Germany) using a T1 image with the following parameters: TR 1900 ms, TE 2.45 ms, voxel size 1.1 × 1.1 × 1.0 mm, slice thickness: 1 mm, and FOV: 272 mm. Additionally, a T2-weighted image was used with the following parameters: TR 3790 ms, TE 100 ms, voxel size 0.4 × 0.4 × 2 mm, slice thickness: 2 mm, FOV: 272 mm, and total acquisition time: 15 min. All cases underwent preoperative CT with fame (Brilliance CT Big Bore, Philips, The Netherlands), and the CT image was reconstructed into 1 mm thick slices with an FOV of 272 mm.

#### 2.2.2. Operation and Intraoperative Macrostimulation Test

Leksell G head frame and Surgiplan system (Elekta AB, Stockholm, Sweden) were adopted. After implanting electrodes under local anesthesia, a bone hole was sealed with biomedical fibrin glue. Then, an external neurostimulator was connected to carry out intraoperative macrostimulation tests and record the threshold value of efficacy and adverse reactions of electrical stimulation. The test cathode contact was the most inferior contact, and the anode contact was the superior contact. The electrodes are 3389 (Medtronic, Villalba, USA) or L301 (PINS, Beijing, China). Stimulation parameters were as follows: pulse width: 60 *μ*s; frequency: 130 Hz; with the voltage increasing from 1.5 V to 5.0 V gradually. Side effects were observed and recorded. After fixing the electrode with Stimloc (Medtronic, Villalba, USA) or Leadloc (PINS, Beijing, China), the scalp was sutured. If the position of the electrode was satisfactory after confirmed by a 1.5 T MRI (Siemens MAGNETOM Avanto, Germany) scanning with frame, an extension lead and implantable pulse generator were implanted under general anesthesia.

#### 2.2.3. Postoperative CT

CT examination was performed within 6 days after operation to exclude intracranial hemorrhage and pneumocephalus and to confirm the position of electrodes and contacts. Postoperative high-resolution CT images acquired in all cases matched preoperative CT.

### 2.3. Position of Electrodes and Contacts

Because of individual specificity of the STN of each patient and in order to unify the position of nuclei and electrodes, postoperative electrode localizations were performed using Lead-DBS software (http://www.lead-dbs.org/) [13]. Postoperative images were linearly coregistered to preoperative images using the Statistical Parametrical Mapping software version 12 (SPM12) [14] and BRAINSFit software [15]. Images were then nonlinearly warped into standard stereotactic (MNI; ICBM152 2009b nonlinear asymmetric) space using a fast diffeomorphic image registration algorithm (DARTEL) [16]. Electrode trajectories were automatically prelocalized, and the results were manually refined in MNI space using Lead-DBS. This procedure allowed us to visualize the recording sites of all cases together in one figure.

Distances between the centers of electrode activated contacts and their nearest voxel of the red nucleus (RN) volume defined on an MNI version [17] of the histology-based Morel atlas [18] were calculated.

### 2.4. Statistical Analysis

Student's *t*-test was used to compare *X* absolute value, *Y* and *Z* coordinates of the center of the lowest contact in MNI space as well as the distances between the centers of electrode activated contacts and their nearest voxel of the red nucleus between the sweating group and the control group. *P* < 0.05 indicated statistical significance.

## 3. Results

### 3.1. Data of Patients

A total of 355 patients were screened, of which 11 patients (18 electrodes) had induced sweat during intraoperative electrical stimulation, including 9 cases of sweats caused by the lowest contact in the left side and 9 cases of sweats caused by the lowest contact in the right side. A total of 11 patients without sweats during intraoperative electronic stimulation were randomly sampled. Difference in baseline data between the two groups was not statistically significant (including gender, age, disease duration, preoperative UPDRS III (Med-on and Med-off), Mini-Mental State Examination (MMSE), Montreal Cognitive Assessment (MoCA), NonMotor Symptom Scale (NMSS), Hamilton Depression Scale item 17 (HAMD), Hamilton Anxiety Scale (HAMA)), as shown in Table 1. Six months after turning the IPG-on and obtaining the optimal programming parameters, no patient in either group had sweats. In addition, the difference in UPDRS III score under IPG-off Med-off, IPG-on Med-off, and IPG-on Med-on between the two groups was not statistically significant (Table 1). A total of 22 patients in the two groups had no intracranial hemorrhage and no hardware-related complication at the one-year follow-up.

### 3.2. Position of Contact Inducing Sweating

**Figure 1:**
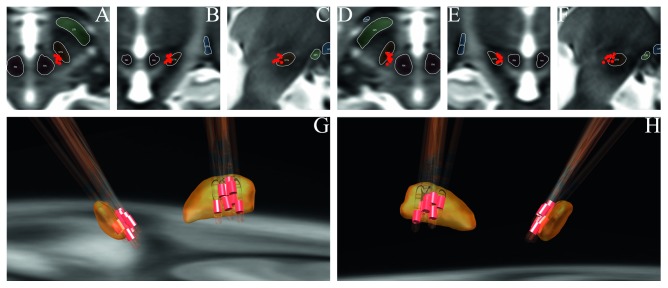
Location of activated cathode contacts of the sweating group. (a) Axial view of left contacts. (b) Coronal view of left contacts. (c) Sagittal view of left contacts. (d) Axial view of right contacts. (e) Coronal view of right contacts. (f) Sagittal view of right contacts. (g) Left-posterior direction view on STN (yellow), electrodes, and activated contacts (red) in 3D. (h) Right-posterior direction view on electrodes and activated contacts (red) in 3D.

**Figure 2:**
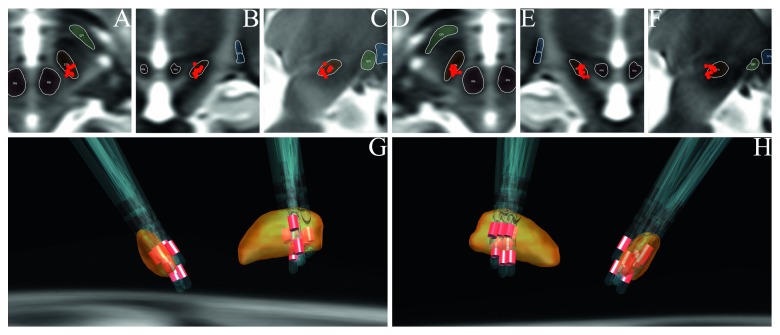
Location of activated cathode contacts of the control group. (a) Axial view of left contacts. (b) Coronal view of left contacts. (c) Sagittal view of left contacts. (d) Axial view of right contacts. (e) Coronal view of right contacts. (f) Sagittal view of right contacts. (g) Left-posterior direction view on STN (yellow), electrodes, and activated contacts (red) in 3D. (h) Right-posterior direction view on electrodes and activated contacts (red) in 3D.

The position of active cathode contacts relative to sweating was calculated by the absolute value of the *X*-axis of coordinates of the two electrode activated contacts. On the basis of comparison of MNI space coordinates of contacts of the two groups, it was found that the contacts inducing sweat were more medial (11.02 ± 0.69 mm VS 11.98 ± 0.84 mm, *P*=0.00057) and more superior (−7.15 ± 1.06 mm VS −7.98 ± 1.21 mm, *P*=0.032) than the control group. Positions of electrodes and activated contacts are shown in Figures 1 and 2. The *Y*-axis showed no difference between the sweating group and control group (−15.44 ± 1.25 mm VS −15.06 ± 1.11 mm). The straight-line distance between the center of contacts of the sweating group and the nearest voxel of the red nucleus was closer than that of the control group (2.72 ± 0.65 mm VS 3.76 ± 0.85 mm, *P*=0.00012).

**Figure 3:**
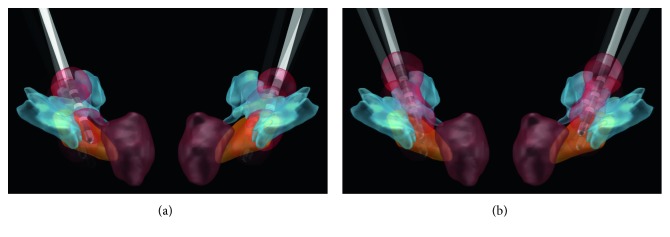
VTA of patients. The VTA of the control group. (a) Only 8 of 22 VTAs stimulated Raprl. (b) Seventeen of 18 VTA stimulated Raprl. STN = orange; ZI = light blue; RN = dark red. Raprl is not shown in the figure.

Unifying patients' imaging data and reconstructing the volume tissue activated (VTA) after electrical stimulation are conducive to understanding scientifically which nerve fibers take part in the process of sweating. However, because patient sweating occurred gradually during macrostimulation tests, it is difficult to accurately confirm the voltage that caused the sweating side effects. Therefore, we set the voltage 5.0 V, frequency 150 Hz, and pulse width 90 *μ*s and reconstructed the VTA of every patients. The result shows (Figure 3) STN and zona incerta (ZI) were stimulated in both groups VTA, but the prelemniscal radiations (Raprl) were mainly stimulated in the sweating group (8/22 vs 17/18, *P*=0.001). The position of Raprl was according to the previous study [19], but Raprl is a structure with a lot of cluster of fibers which could not be shown in its entirety in Lead-DBS and Figure 3.

## 4. Discussion

### 4.1. STN-DBS-Induced Sweating Only Related to Contact Position

The study indicated that patients' preoperative baseline data (Table 1) did not have obvious predictive value for sweating induced by postoperative electronic stimulation. The main reason for sweating was the anatomical position of activated contacts.

### 4.2. STN-DBS-Induced Sweating Did Not Impact the Patient's Motor Symptoms Relieve

Previous studies show that STN and surrounding structures participate in sudomotor dysfunction. STN-DBS can improve the symptom of hyperhidrosis [20–24] in some patients, but stimulating the STN or PSA [7–9] could also result in sweating. In addition, when the electrode is close to the hypothalamus, electrical stimulation can also induce sweat [25]. While stimulation-induced hyperhidrosis has little effect on most patients' quality of life, it remains an interesting scientific issue.

### 4.3. Brain Structures Might Involve STN-DBS-Induced Sweating

Data of the study indicated that the position of contacts inducing hyperhidrosis was slightly more anterior than that in the PSA region. Contacts located in the MNI space such that *X-*axis: 11.02 ± 0.69, *Y*-axis: −15.44 ± 1.25, and *Z*-axis: −7.15 ± 1.06. In front of this area is the lateral hypothalamic (LH) area, H2 area is above the area, ZI is above H2 area, and capsule of the red nucleus is medial to the area [26]. And, tracts from Raprl to orbitofrontal cortex (OFC, terminal cortex including lateral and medial orbitofrontal cortex) and to prefrontal cortex (PFC, which comprises the frontal pole, pars orbitalis, pars triangularis, and rostral middle frontal gyrus) [19] also pass through the area. This finding supports that ZI [7, 27] or fibers from the hypothalamic paraventricular nucleus [28] might lead to sweat.

Although we could not confirm which structure is responsible for STN-DBS-induced sweating, we suggest addition of the Raprl-OFC and Raprl-PFC as suspected criminal structure for the following reasons: (1) PSA contains Raprl-OFC and Raprl-PFC tracts [19] and could also induced sweating by DBS [8, 9]; (2) this study found more Raprl areas (including Raprl-OFC and Raprl-PFC tracts) activated by VTA in the sweating group; (3) electrical stimulation-induced sweating can also be observed [29] during treating mental disorder with DBS in nucleus accumbens and anterior limb of internal capsule [25, 29]. Moreover, the Raprl-PFC and Raprl-OFC pass through the nucleus accumbens and anterior limb of internal capsule [19]. But, more research and evidence are necessary to establish definitively whether Raprl-OFC and PFC fibers participate in DBS-induced sweating.

### 4.4. Clinical Value of STN-DBS-Induced Sweating during Operation

From the clinical perspective, sweating induced by intraoperative tests may indicate that the lowest contact is at the medial part of STN or close to the red nucleus. It may not influence the improvement in patients' motor symptoms by electronical stimulation after operation, because the contact finally selected for programming was close to the dorsolateral part of STN rather than being the lowest contact. However, the symptom of sweating is of certain guiding phenomenon, and neurosurgeons should consider whether the Pitch trajectory angle of the electrode is too large or the electrode placement is too medial. Macrostimulation tests should be carried out seriously to observe and determine whether patients have numbness of their extremities or other manifestations that may be induced by RN stimulation, which might be helpful for adjusting the electrode position during the operation.

Interestingly, while most patients sweated from the ipsilateral skin after electrical stimulation, in other patients, the contralateral skin also sweated after a period of time. This indicates that bilateral sweating structures may be synergetic, similar to the STN [30].

## 5. Conclusions

Sweating caused by STN-DBS indicates that the contacts are close to the medial edge of the STN.

## Figures and Tables

**Table 1:** Preoperative baseline data of patients and data of motor symptoms during 6 months of power-on.

Groups	Sweating group	Normal group	*P*
Gender	5F (6M)	4F (7M)	1.000
Age	62.54 ± 7.63	63.08 ± 9.24	0.398
Disease duration	8.85 ± 2.48	10.54 ± 4.93	0.160
UPDRS-III (Med-off before operation)	61.38 ± 11.79	58.92 ± 19.76	0.746
UPDRS-III (Med-on before operation)	30.46 ± 10.15	30.85 ± 14.00	0.487
MMSE	28.00 ± 1.87	27.38 ± 1.89	0.720
MoCA	25.00 ± 3.37	25.85 ± 2.48	0.427
NMSs	18.15 ± 2.54	18.85 ± 4.51	0.277
HAMD	17.54 ± 3.97	18.92 ± 5.77	0.404
HAMA	14.85 ± 2.88	15.31 ± 3.59	0.457
UPDRS-III (Med-off IPG-off 6 m after operation)	57.08 ± 16.06	57.38 ± 20.83	0.564
UPDRS-III (Med-off IPG-on 6 m after operation)	33.77 ± 9.75	29.46 ± 11.52	0.946
UPDRS-III (Med-on IPG-on 6 m after operation)	24.23 ± 8.20	23.08 ± 6.61	0.955

## Data Availability

The data used to support the findings of this study are available from the corresponding author upon request.
